# The effects of Xuebijing injection combined with ulinastatin as adjunctive therapy on sepsis: An overview of systematic review and meta-analysis

**DOI:** 10.1097/MD.0000000000031196

**Published:** 2022-10-21

**Authors:** Guofu Zhong, Yue Han, Qinghua Zhu, Mujuan Xu, Xiao Chang, Mingtai Chen, Ling Men, Qiang Zhang, Ling Wang

**Affiliations:** a Shenzhen Traditional Chinese Medicine Hospital, Shenzhen, China.

**Keywords:** efficacy and safety, overview, sepsis, systematic review, Xuebijing injection

## Abstract

Xuebijing injection (XBJ) has increasingly been used for sepsis in China. We aimed to evaluate the methodological quality and summarize the evidence regarding the effectiveness of XBJ combined with ulinastatin (UTI) for sepsis from systematic reviews/meta-analyses (SRs/MAs).

**Methods:**

From the inception to May 23, 2021, eight databases were searched to screen the SRs/MAs of XBJ combined with UTI in the treatment of sepsis. Methodology Quality of Systematic Reviews 2 (AMSTAR-2) was used to evaluate the quality of the methods. Grading of Recommendation,Assessment, Development, and Evaluation (GRADE) was used in the assessment of evidence quality.

**Results:**

Seven SRs/MAs on XBJ combined with UTI treatment for sepsis were included. The AMSTAR-2 showed that the methodological quality of all included SRs/MAs was rated as critically low. According to the evaluation results of GRADE, 30% (13/44), 30% (13/44), and 40% (18/44) were rated to be of moderate, low, and critically low quality, respectively. Descriptive analysis results showed that XBJ combined with UTI was an effective treatment modality for sepsis.

**Conclusions:**

All included SRs/MAs showed that XBJ combined with UTI was more effective than UTI alone in the treatment of sepsis on the basis of conventional treatment, but the reliability of the results was limited due to the disadvantages of lower methodological quality and higher risk of bias in the included SRs/MAs. Further high-quality clinical studies and SRs/MAs are recommended to verify whether XBJ combined with UTI is more effective than UTI alone.

## 1. Introduction

Sepsis is a life-threatening organ dysfunction caused by the dysregulation of the host’s response to infection, accompanied by high morbidity, mortality and treatment costs, and is a major global health problem.^[1]^ In the United States, for example, sepsis is the most common cause of death in hospitals, costing more than $24 billion annually.^[2]^ Epidemiological studies show that in 2017, the number of sepsis cases in the world was 489 million, including 11 million deaths.^[3]^ Despite great progress in clinical treatment of sepsis in the past decades, there is still a high rate of intensive care unit (ICU) admission, which is the main cause of death in many ICU units.^[4]^ In addition, some studies have reported that the incidence and burden of sepsis are still increasing year by year, seriously affecting the quality of life of patients.^[5]^ So far, the preventive measures, specific drugs, and management strategies of modern medical treatment to control sepsis are quite limited.

Xuebijing injection (XBJ) is a water-soluble intravenous injection made from the extracts of 5 traditional Chinese medicines: Rhizoma Chuanxiong, Radix Angelicae Sinensis, Radix Salviae Miltiorrhizae, Radix Paeoniae Rubra, and Flos Carthami. XBJ is based on the ancient Chinese prescription “Xuefu Zhuyu Decoction”, which has the effects of promoting blood circulation to remove blood stasis, relaxing the collaterals and dissipating toxic evil.^[6]^ Pharmacological studies have shown that XBJ is an effective drug to improve the survival rate by blocking the progression of sepsis through antimicrobial, anti-inflammatory and anti-endotoxin.^[7]^ In recent years, XBJ, as the only Chinese patent medicine approved for the treatment of sepsis in China’s clinical guidelines, has been proved by many clinical trials and systematic reviews/meta analyses (SRs/MAs) to have a good clinical effect of XBJ combined with ulinastatin (UTI) in the treatment of sepsis.^[8]^ However, the methodological quality of a single study will affect the final evidence quality and grading to some extent, and then affect the true degree of efficacy of XBJ, because there is no literature report to evaluate the methodological and evidence quality of SRs/MAs of XBJ with UTI in the treatment of sepsis. Therefore, this study conducted a summary analysis and evaluation of the existing SRs/MAs and their outcome indicators to provide further evidence-based support for the clinical use of XBJ.

## 2. Materials and Methods

### 2.1. Inclusion and exclusion criteria

Inclusion criteria were as follows: study design: English and Chinese literature on SRs/MAs based on randomized controlled trials (RCTs); participants: participants have been diagnosed with sepsis according to any authoritative diagnostic criteria with no restrictions on gender, age or race; intervention: Both the experimental group and the control group received conventional anti-septic treatment. Based on this treatment, the experimental group was given XBJ combined with UTI, and the control group was given UTI or XBJ alone; and outcomes: duration of mechanical ventilation, length of ICU stays, 28-day mortality, acute physiology and chronic health evaluation (APACHE) II score, multi-organ dysfunction syndrome (MODS), serum levels of inflammatory cytokines, procalcitonin (PCT) and lipopolysaccharide (LPS).

The exclusion criteria were as follows: documents that are published repeatedly; documents whose full text is not available; documents with incomplete data.

### 2.2. Search strategy

CNKI, Wanfang, VIP, CBM, Embase, PubMed, Cochrane Library, Web of Science electronic databases were searched by computer. Two major clinical trial registration systems, ClinicalTrials.gov and China Clinical Trial Registry, were consulted to collect literatures on XBJ combined with UTI in the treatment of sepsis in both Chinese and English. The retrieval time is from the establishment to May 2021. Supplemental digital content 1, http://links.lww.com/MD/H656 provides detailed information about the search strategy of each database.

### 2.3. Eligibility assessment and data extraction

Two researchers independently screened the literature, extracted the data, and cross-checked it. If there are differences, they shall be resolved through discussion or negotiation with a third researcher. During literature screening, the title and abstract were first read, and the full text was further read to determine inclusion after excluding the literature that was significantly irrelevant and did not meet the inclusion criteria. If necessary, contact the original study author via email or phone for necessary information. Data extraction included author, publication time, number of included studies, number of included samples, publication time span of included studies, intervention measures, outcome indicators and main outcome indicators, bias risk assessment tools, publication journals, main conclusions, etc.

### 2.4. Methodological quality assessment

The methodological quality of the included literature was assessed using the AMSTAR-2 tool.^[9]^ Amstar-2 tool has a total of 16 items, of which items 2, 4, 7, 9, 11, 13, and 15 are the key items. Each item can be rated as “Yes”, “Partial Yes”, “No”, and “No Meta-analysis”. Specific quality evaluation criteria are as follows: no or only 1 non-critical item does not meet the evaluation as high quality; More than 1 non-critical item does not meet the evaluation as medium quality; 1 key item is not conformed and accompanied by or without non-key item is not conformed to the evaluation as low quality; More than 1 critical item is not conformed, with or without non-critical item is not conformed to the evaluation of very low quality.

### 2.5. GRADE scoring

The GRADE tool^[10]^ was used to evaluate the quality for the included literature outcome indicators. The level of evidence for outcome indicators was evaluated from five aspects (risk of bias, inconsistency, indirectness, inaccuracy, and publication bias). Grade divides evidence quality into: high quality (confident that effect estimates of the amount of close to the real situation), (medium quality, it is possible that the true value is close to the estimated value, but there is still the possibility that the two are very different), low quality (confidence in the estimate of effect size is limited, may have great difference with the real situation), very low quality (very little confidence in the estimate of effect size, May be very different from the real situation). Results summary tables were used to summarize the evidence quality evaluation results and the reasons for the downgrade for each included study.

## 3. Results

### 3.1. Literature selection

**Figure 1.**
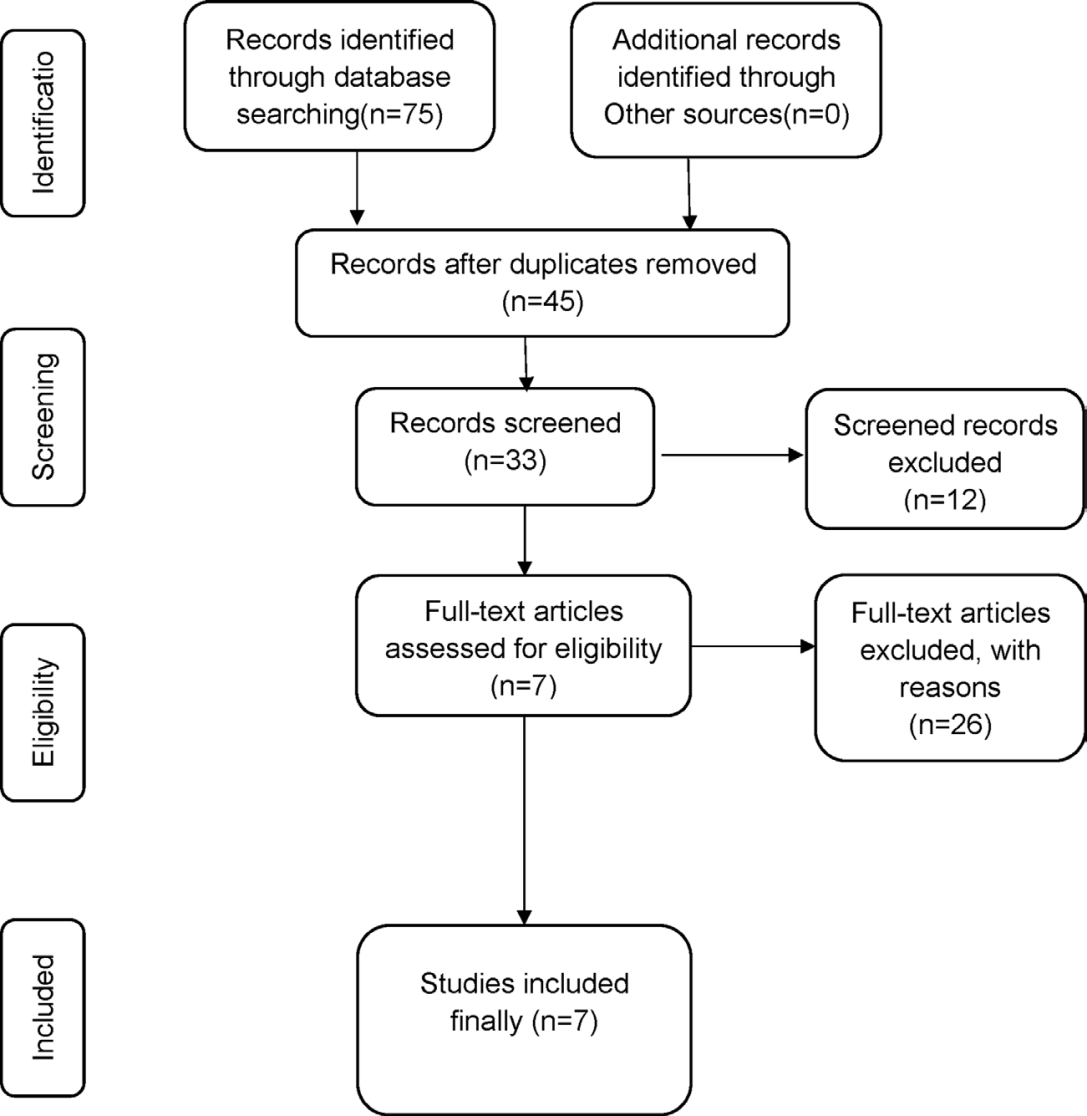
Flow diagram of the literature selection process.

A total of 75 studies were retrieved, of which 45 remained after duplication was removed, and 12 unrelated studies were excluded by reading the titles and abstracts. By reviewing the full text of the remaining 33 studies, 2 duplicates and 24 studies that did not provide results of XBJ injection were further excluded, and finally 7 systematic reviews were included. The research flow chart is shown in Figure 1.

### 3.2. Study characteristics

Seven systematic reviews^[11–17]^ were included, including 2^[11]^ in English and 5^[12]^ in Chinese, published from 2014 to 2018. The included studies were all RCTs, the number of included studies ranged from 4 to 17, and the intervention in the trial group was XBJ combined with UTI. The intervention measures in the control group were UTI or XBJ alone. Methodological evaluation was performed on the included original studies, 3^[13]^ of which used the risk bias assessment tool recommended by the Cochrane Systematic Rater’s Manual, and 4^[14]^ of which used the Jadad scale. Table 1 for the basic characteristics of the specific studies.

**Table 1:** Characteristics of the included reviews.

Studies	Country	Language	Trials (sample size)	Treatment intervention	Control intervention	Quality assessment tool
Li et al 2014	China	Chinese	4 (362)	CM+XBJ+UTI	CM+UTI	Jadad
Sui et al 2017	China	Chinese	16 (1209)	CM+XBJ+UTI	CM+UTI; CM+XBJ	Jadad
Jiang et al 2015	China	Chinese	15 (1222)	CM+XBJ+UTI	CM+UTI; CM+XBJ	Jadad
Liao et al 2014	China	Chinese	8 (735)	CM+XBJ+UTI	CM+UTI; CM+XBJ	Jadad
Zhou et al 2014	China	Chinese	15 (1469)	CM+XBJ+UTI	CM+UTI; CM+XBJ	Cochrane criteria
Chen et al 2018	China	English	17 (1247)	CM+XBJ+UTI	CM+UTI	Cochrane criteria
Xiao et al 2018	China	English	16 (1335)	CM+XBJ+UTI	CM+UTI	Cochrane criteria

CM = conventional medication, UTI = ulinastatin, XBJ = Xuebijing.

### 3.3. Methodological evaluation

The AMSTAR-2 scale was used to evaluate the methodological quality of the included systematic evaluations. Among the 7 SRs/MAs, only 2 articles failed to meet the requirements of 1 key item, and combined with multiple non-key items, the methodological quality was low, while the other 5 articles failed to meet the requirements of more than 1 key item. The combination of multiple non-critical entries is not met and the methodological quality is extremely low. None of the literatures mentioned the formulation of the research protocol, none of the six literatures searched the trial registry, failed to search the literatures comprehensively, and one of the literatures failed to describe that the literature screening and data extraction were completed by two independent reviewers. 3 articles not specified in the selection flow chart after reading the full text eliminate the reason of the article 1 article did not provide a document listing, 1 piece of literature did not provide a table into the research characteristics, 1 piece of literature did not describe the dose of interventions, 1 piece of literature information describing the study population, not 1 articles were not carried out on the original research bias risk assessment, The quality evaluation of 1 literature was incomplete, the cause analysis and treatment of heterogeneity were not carried out in 1 literature, and the conflict of interest was not reported in 5 Chinese literatures, as shown in Table 2.

**Table 2:** Results of the AMSTAR-2 assessments.

Studies	AMSTAR-2																Overall quality
	Q1	Q2	Q3	Q4	Q5	Q6	Q7	Q8	Q9	Q10	Q11	Q12	Q13	Q14	Q15	Q16	
Li et al 2014	Y	N	Y	PY	N	N	N	Y	Y	N	PY	PY	PY	Y	Y	N	CL
Sui et al 2017	Y	N	Y	PY	Y	Y	N	Y	Y	N	Y	Y	Y	Y	Y	N	CL
Jiang et al 2015	Y	N	Y	PY	Y	Y	Y	Y	Y	Y	Y	Y	Y	Y	Y	N	CL
Liao et al 2014	Y	N	Y	PY	Y	Y	Y	Y	Y	N	Y	Y	Y	Y	Y	N	CL
Zhou et al 2014	Y	N	Y	PY	Y	Y	N	N	Y	N	Y	Y	Y	N	N	N	CL
Chen et al 2018	Y	N	Y	PY	Y	Y	Y	Y	Y	Y	Y	Y	Y	Y	Y	Y	L
Xiao et al 2018	Y	N	Y	Y	Y	Y	Y	Y	Y	Y	Y	Y	Y	Y	Y	Y	L
Y (%)	100	0	100	14	86	86	43	71	100	43	86	86	86	86	100	29	

CL = critically low, H = high, L = low, N = no, PY = partial yes, Y = yes.

### 3.4. Evidence quality evaluation

A total of 44 outcomes indicators of Meta-analysis were applied in the included SRs/MAs, and GRADE system was used to evaluate the quality of each outcome indicator. The results showed that 30% (13/44), 30% (13/44), and 40% (18/44) were rated to be of moderate, low, and critically low quality, respectively. The details of the evaluation are shown in Table 3.

**Table 3:** Certainty of evidence quality evaluation.

Studies	Interventions	Outcomes	Studies (participants)	Limitations	Inconsistency	Indirectness	Imprecision	Publication bias	Quality
Chen et al 2018	XBJ+UTI+CT vs UTI+CT	28-d mortality	6 (597)	−1*	0	0	0	0	Moderate
		Duration of mechanical ventilation	8 (578)	−1*	0	0	0	0	Moderate
		Length of ICU stay	9 (618)	−1*	0	0	0	0	Moderate
		APACHE II score	7 (482)	−1*	−1†	0	0	0	Low
		PCT	8 (668)	−1*	−1†	0	0	0	Low
		LPS	3 (288)	−1*	0	0	−1‡	−1§	Very low
Xiao et al 2018	XBJ+UTI+CT vs UTI+CT	Duration of mechanical ventilation	8 (556)	−1*	0	0	0	0	Moderate
		28-d survival rate	5 (428)	−1*	0	0	0	0	Moderate
		Length of ICU stay	9 (676)	−1*	0	0	0	0	Moderate
		APACHE II score	7 (662)	−1*	−1†	0	0	0	Low
		PCT	7 (832)	−1*	−1†	0	0	0	Low
		Occurrence rate of MODS	2 (136)	−1*	0	0	−1‡	1§	Very low
		TNF	6 (428)	−1*	−1†	0	0	0	Low
		IL-6	8 (480)	−1*	−1†	0	0	0	Low
		Case fatality rate	3 (136)	−1*	0	0	−1‡	1§	Very low
Li et al 2014	XBJ+UTI+CT vs UTI+CT	Duration of mechanical ventilation	4 (328)	−1*	0	0	−1‡	1§	Very low
		IL-6	4 (410)	−1*	0	0	−1‡	1§	Very low
		Length of ICU stay	4 (328)	−1*	0	0	−1‡	1§	Very low
		TNF	4 (410)	−1*	0	0	−1‡	1§	Very low
		PCT	4 (410)	−1*	−1†	0	−1‡	1§	Very low
Sui et al 2017	XBJ+UTI+CT vs UTI+CT	Duration of mechanical ventilation	5 (440)	−1*	−1†	0	0	0	Low
		Length of ICU stay	5 (411)	−1*	0	0	0	0	Moderate
		APACHE II score	10 (726)	−1*	−1†	0	0	0	Low
		PCT	9 (679)	−1*	−1†	0	0	0	Low
		Occurrence rate of MODS	3 (204)	−1*	0	0	−1‡	1§	Very low
		Case fatality rate	11 (741)	−1*	0	0	0	0	Moderate
		LPS	3 (695)	−1*	0	0	−1‡	1§	Very low
		TNF	6 (695)	−1*	0	0	0	0	Moderate
Zhou et al 2014	XBJ+UTI+CT vs UTI+CT	Duration of mechanical ventilation	4 (NA)	−1*	−1†	0	−1‡	1§	Very low
		Length of ICU stay	5 (NA)	−1*	−1†	0	0	0	Low
		APACHE II score	7 (NA)	−1*	−1†	0	0	0	Low
		PCT	3 (NA)	−1*	−1†	0	−1‡	1§	Very low
		Case fatality rate	3 (NA)	−1*	−1†	0	−1‡	1§	Very low
		LPS	3 (NA)	−1*	−1†	0	−1‡	1§	Very low
Liao et al 2014	XBJ+UTI+CT vs UTI+CT	Duration of mechanical ventilation	5 (358)	−1*	0	0	0	0	Moderate
		Length of ICU stay	5 (358)	−1*	0	0	0	0	Moderate
		APACHE II score	2 (175)	−1*	0	0	−1‡	1§	Very low
		PCT	4 (396)	−1*	0	0	−1‡	1§	Very low
		IL-6	4 (396)	−1*	−1†	0	−1‡	1§	Very low
		LPS	2 (175)	−1*	0	0	−1‡	1§	Very low
Jiang et al 2015	XBJ+UTI+CT vs UTI+CT	Duration of mechanical ventilation	6 (647)	−1*	0	0	0	0	Moderate
		Length of ICU stay	7 (446)	−1*	−1†	0	0	0	Low
		APACHE II score	9 (647)	−1*	−1†	0	0	0	Low
		Case fatality rate	9 (579)	−1*	0	0	0	0	Moderate

APACHE = acute physiology and chronic health evaluation, CM = conventional medication, ICU = intensive care unit, IL-6 = interleukin 6, LPS = lipopolysaccharide, MODS = multi-organ dysfunction syndrome, NA = not reported, PCT = procalcitonin, TNF = tumor necrosis factor, UTI = ulinastatin, XBJ = Xuebijing.

*The design of the experiment has a large bias in randomization, distributive concealment, or blinding.

†The confidence interval overlaps less, the heterogeneity test *P* is very small, and the *I*^2^ is larger.

‡The confidence interval is not narrow enough.

§Funnel graph asymmetry; fewer studies are included and there may be a greater risk of publication bias.

### 3.5. Outcomes and efficacy evaluation

#### 3.5.1. 28-day mortality.

Two researches^[16,17]^ compared mortality within 28 days between XBJ plus UTI group and single UTI group. The final data showed that XBJ combined with UII significantly reduced 28-day mortality.

#### 3.5.2. Duration of mechanical ventilation.

All SRs/MAs^[11–17]^ compared duration of the mechanical ventilation between XBJ plus UTI group and single UTI group. Clinical studies have shown that XBJ combined with UII has less duration of mechanical ventilation than UII alone.

#### 3.5.3. Length of ICU stay.

All SRs/MAs^[11–17]^ compared the length of ICU stay between XBJ plus UTI group and single UTI group. It was found that XBJ plus UTI can significantly reduce the length of stay in ICU.

#### 3.5.4. APACHE II score.

6 SRs/MAs^[11–17]^ compared the APACHE II score between XBJ plus UTI group and single UTI group. It was found that XBJ combined with UTI was superior to single UTI in terms of ameliorating APACHE II score.

#### 3.5.5. Occurrence rate of MODS.

2 SRs/MAs^[14,17]^ compared the occurrence rate of MODS between XBJ plus UTI group and single UTI group. The results showed that the combination of XBJ plus UTI significantly reduced the incidence of MODS compared with UTI alone.

#### 3.5.6. PCT.

6 SRs/MAs^[12–17]^ compared the PCT between XBJ plus UTI group and single UTI group. The results mentioned above suggested that the combination of XBJ and UTI was superior to UTI alone in reducing serum PCT levels.

#### 3.5.7. LPS.

4 SRs/MAs^[13–16]^ compared the LPS between XBJ plus UTI group and single UTI group. It was found that a combination of XBJ and UTI could significantly lower LPS levels than single UTI.

#### 3.5.8. IL-6.

3 SRs/MAs^[12,13,17]^ compared the interleukin 6 (IL-6) between XBJ plus UTI group and single UTI group. The pooled analysis manifested that compared to single UTI, a combination of XBJ and UTI was more effective in lowering IL-6 level.

#### 3.5.9. TNF.

3 SRs/MAs^[12,13,17]^ compared the tumor necrosis factor (TNF) between XBJ plus UTI group and single UTI group. According to the results, it was signified that XBJ combined with UTI decreased TNF levels in a greater degree than UTI alone.

#### 3.5.10. Case fatality rate.

3 SRs/MAs^[14,15,17]^ compared the TNF between XBJ plus UTI group and single UTI group. It indicated that when comparing to UTI, XBJ plus UTI was more effective in improving case fatality rate.

## 4. Discussion

SRs/MAs is at the top of the evidence quality grading pyramid of evidence-based medicine, and is recognized as the cornerstone for evaluating clinical efficacy and formulating clinical guidelines and norms.^[18,19]^ Systematic evaluation reevaluation is a comprehensive research method to collect and reevaluate the related systematic evaluation of the treatment and diagnosis of the same disease or health problem comprehensively, which can provide more concentrated and high-quality evidence for the evidence users.^[19]^ In this study, the systematic review/meta-analysis of XBJ combined with UTI in the treatment of sepsis was reevaluated, and the methodological quality of the included studies was evaluated using the AMSTAR-2 scale, and the evidence quality of the included studies was evaluated using the GRADE system. This study provides high-quality evidence-based evidence and decision-making basis for the treatment of sepsis with XBJ combined with UTI, and further provides clinical reference.

### 4.1. Summary of quality

The results of the AMSTAR-2 evaluation indicate that there are serious methodological deficiencies in all SRs/MAs included in this study. First, none of the studies had a research proposal. Domestic researchers do not pay enough attention to the early registration of systematic evaluation, which leads to the lack of open and visible protocols for the research, affecting its transparency and credibility. Public registration information can be obtained in advance, which will help researchers avoid post-decision bias in the process of system evaluation. Researchers are encouraged to register the proposal on relevant platforms before conducting systematic evaluation, to improve the scientific nature and transparency of the research. Secondly, there is a general lack of comprehensive and standard retrieval and screening.^[20]^ Retrieval of gray literature, clinical trial registration platform and other data other than electronic databases is of great significance for the control of publication bias,^[21]^ but has been neglected by researchers. The report on the complete retrieval strategy and the exclusion list is the weak link that the domestic and foreign researchers need to strengthen in the future. Third, the necessary information is not described. Data extraction from included studies is the primary preparatory work for SRs/MAs, while demographic information, dosage form and dose of intervention, observation time point, and efficacy evaluation criteria are all important basis for judging clinical heterogeneity between studies, and the main reasons for statistical heterogeneity are explained. In order to avoid unnecessary heterogeneity and enhance the credibility of quantitative synthesis effect values, researchers should extract and record relevant information as comprehensively as possible. In addition, insufficient heterogeneity analysis. All researchers can simply judge the magnitude of statistical heterogeneity through *I*^2^ and *P*, and provide corresponding models. However, the treatment of high heterogeneity is a methodological blind spot for many researchers. The research team for the systematic review must include methodological experts. Based on strengthening multidisciplinary cooperation, the quality of systematic evaluation should be improved to promote the development of evidence-based medicine.^[22]^ In the end, no indication of conflict of interest. Studies have found that systematic evaluations with conflicts of interest are more likely to draw favorable conclusions than those without conflicts of interest, and the methodological quality is often lower. Systematic evaluation without conflict of interest is recommended in the development of clinical guidelines. Therefore, researchers should pay attention to the transparency of funding sources and conflicts of interest and standardize related items in reports.

According to GRADE’s evaluation results, the quality of evidence is between moderate and very low, with only 13 results rated as moderate, 13 results rated as low, and the remaining 18 results rated as very low. The lower the quality, the more likely it is that further research will change our confidence in the estimates themselves. Therefore, caution should be exercised when recommending XBJ as an alternative therapy for the treatment of sepsis. The most common downgrade factors were the risk of bias, inconsistency, inaccuracy, and the possibility of publication bias in the original trial. Of most of these SRs/MAs included in RCTs, their methodological quality was low. The drawbacks of the consistent approach are as follows: RCTs are described as random and do not provide a method for random sequence generation; Most RCTs do not explicitly state that treatment allocation is hidden; Most randomized controlled trials fail to blind subjects and doctors. These findings suggest that there is still a lot of room to address method quality issues in the RCT process. High-quality RCT with large sample size should be the focus of future studies on XBJ.

In recent years, a series of in vivo and in vitro studies have revealed the mechanism of action of XBJ in the treatment of sepsis. In a mouse model of sepsis induced by cescal ligation and puncture surgeries,^[23]^ it has been demonstrated that XBJ improves survival in septic mice by preventing cytokine storm and inhibiting serum levels of the inflammatory cytokines TNF-α and IL-6.^[23]^ In addition, it has also been demonstrated that XBJ significantly improves survival and rescues cardiac insufficiency in vivo in septic mice, mainly by inhibiting the expression of pro-inflammatory cytokines/chemokines and related pathways in cardiac tissue.^[24]^ Interestingly, the five botanicals that constitute XBJI have been reported to be closely associated with sepsis or anti-inflammatory responses.^[25]^ According to TCM theory, XBJ could clear heat and detoxify blood and activate blood stasis. For example, among the phytomedicinal components of XBJ, *Radix Salviae Miltiorrhizae* attenuates endotoxin-induced HMGB1 release from macrophages and monocyte cultures, which is a late mediator of lethal sepsis.^[26]^ In the LPS-induced RAW 264.7 cell model, the main components of *Radix Paeoniae Rubra*, paeoniflorin and paeoniflorin, were found to have anti-inflammatory activity.^[27]^

### 4.2. Limitations

There are still some limitations in this study. For example, only Chinese and English literature databases were retrieved in this study, so relevant literatures may be missed. There were differences in the efficacy evaluation criteria and intervention methods adopted in the included literatures, and all outcome indicators were not quantitatively combined and analyzed. The results of the original literatures were only summarized and reported in the form of tables, and the clinical efficacy of XBJ could not be accurately evaluated.

## 5. Conclusion

In conclusion, XBJ in combination with UTI may be more beneficial for sepsis patients than UTI alone. However, physicians should use evidence in clinical practice to determine issues regarding the use of XBJ to prudently treat sepsis because of the generally low methodologic quality and quality of evidence including SRs/MAs.

## Author contributions

GZ and YH should be considered as co-first authors who have contributed equally to this work.

LW plays the role of guarantor for this manuscript. The manuscript draft was prepared by GZ and YH. QZ, XC, and MX developed the search strategy. MC and LM independently extracted data and screen the potential studies against eligibility criteria. Data synthesis and assess the risk bias of studies will be performed by XC and QZ. LW will ensure that no errors occur and arbitrate any disagreement during the review. All authors reviewed and approved the final version of the manuscript.

**Data curation:** Mujuan Xu.

**Formal analysis:** Mingtai Chen, Ling Men.

**Methodology:** Qinghua Zhu.

**Project administration:** Ling Wang.

**Validation:** Qiang Zhang.

**Writing – original draft:** Guofu Zhong.

**Writing – review & editing:** Yue Han.

## Supplementary Material


