# Supplementary material for: The effects of Xuebijing injection combined with ulinastatin as adjunctive therapy on sepsis: An overview of systematic review and meta-analysis
Source: Medicine (Baltimore). 2022 Oct 21;101(42):e31196. doi: 10.1097/MD.0000000000031196 (PMC9592478; doi:10.1097/MD.0000000000031196)
Supplement: Supplementary file 1 [file medi-101-e31196-s001.pdf]

Supplemental digital content 1

Searching Strategies on PubMed

#1. systematic review [mesh]
#2. review [tiab]
#3. meta-analysis [mesh]
#4. systematic review [tiab]
#5. meta-analysis [tiab]
#6. meta analysis [tiab]
#7. Overview, Clinical Trial [tiab]
#8. Clinical Trial Overview [tiab]
#9. Xuebijing Injection [tiab]
#10. Sepsis [tiab]
#11. 1 or 2 or 3 or 4 or 5 or 6 or 7 or 8
#12. 10 and 11 and 12

Searching Strategies on CNKI:

SU('脓毒症') AND SU 血必净 AND SU('系统综述'+'系统评价'+'系统'+'Meta 分析'+'荟萃分析'+'汇总分析'+'集成分析'+'二次分析'+'衍生分析')
